# Proteostasis breakdown and aggregate diversity in aging and neurodegenerative disease

**DOI:** 10.3389/fnagi.2026.1910154

**Published:** 2026-08-11

**Authors:** Zohaib Nisar Khan

**Affiliations:** Department of Radiation Oncology, Weill Cornell Medicine, New York, NY, United States

**Keywords:** aging, amyloid-β and tau, glymphatic system, neurodegenerative disease, protein aggregation, proteostasis

## Abstract

Protein aggregation and proteostasis decline are central features of aging and neurodegenerative disease, arising from progressive impairment of protein quality-control systems and transitions into aggregation-prone states. During aging, diverse proteins, including metabolic and proteostasis-related factors, gradually accumulate in aggregated forms as proteostasis capacity declines. Although these aggregates often remain compatible with cellular function, increasing aggregate burden progressively challenges proteostasis resilience. Neurodegenerative diseases are characterized by the emergence and amplification of highly toxic, structurally ordered protein assemblies, including amyloid-β (Aβ) and tau, which exist along a continuum with broader age-associated proteome instability rather than as entirely distinct phenomena. Oxidative and metabolic imbalances promote non-enzymatic post-translational modifications (PTMs), including cysteine oxidation, S-nitrosylation, and carbonylation, which alter protein structure, impair degradation pathways, and facilitate misfolding and aggregation. Here, we compare oxidative PTM-driven aggregates observed during aging with disease-associated protein assemblies, highlighting both shared biophysical mechanisms and distinct pathological outcomes. We propose a threshold model in which the cumulative burden of oxidative PTMs, protein misfolding, and impaired clearance progressively erodes proteostasis capacity, increasing susceptibility to the emergence of selective, self-amplifying aggregates. By integrating intracellular quality-control systems with extracellular clearance pathways, including glymphatic and meningeal lymphatic networks, this framework provides a mechanistic perspective on how aggregate diversity evolves toward disease-associated pathology and suggests therapeutic strategies that combine aggregate-specific targeting with restoration of global proteostasis and clearance capacity.

## 1 Introduction

Protein homeostasis (proteostasis) is fundamental for neuronal health and longevity. In neurons, proteostasis is maintained by an integrated network of molecular chaperones, the ubiquitin–proteasome system (UPS), and the autophagy–lysosome pathway (Hipp et al., 2019; Wilson et al., 2023; Yao and Shen, 2023). The UPS primarily degrades soluble and short-lived proteins through ubiquitin-dependent proteasomal degradation, whereas autophagic pathways, including macroautophagy, microautophagy, and chaperone-mediated autophagy (CMA), mediate lysosomal clearance of bulk cytoplasmic components, damaged organelles, and aggregated proteins (Yao and Shen, 2023). These quality-control mechanisms are particularly important in long-lived, post-mitotic neurons, which cannot dilute damaged proteins through cell division (Kinger et al., 2024; Hipp et al., 2019; Labbadia and Morimoto, 2015).

With advancing age, the efficiency of these proteostatic systems declines systemically. This age-dependent breakdown impairs the cell’s ability to triage damaged or misfolded proteins, leading to their gradual accumulation as non-functional aggregates (Höhn et al., 2020). While healthy aging is often accompanied by widespread subclinical protein aggregation, encompassing metabolic enzymes, structural elements, and even components of the proteostasis machinery itself, this process often remains compatible with cellular function and does not necessarily lead to overt neurodegenerative phenotypes (Cuanalo-Contreras et al., 2023; David, 2012). In contrast, neurodegenerative diseases such as Alzheimer’s disease (AD), Parkinson’s disease (PD), amyotrophic lateral sclerosis (ALS), and Huntington’s disease (HD) are defined by the accumulation of highly toxic, structurally ordered oligomeric and fibrillar assemblies, most notably amyloid-β, tau, α-synuclein, TDP-43, and huntingtin (Chiti and Dobson, 2017; Knowles et al., 2014; Kinger et al., 2024; Wilson et al., 2023).

Aging-related oxidative stress further exacerbates this proteostatic decline by promoting a dense spectrum of non-enzymatic, oxidative post-translational modifications (PTMs). Most notably, cysteine oxidation, S-nitrosylation, and carbonylation act alongside altered enzymatic signaling (such as aberrant phosphorylation and ubiquitination) to destabilize native protein conformations and disrupt essential protein-protein interactions (Höhn et al., 2020; Wilson et al., 2023). Importantly, oxidative PTMs exert context-dependent effects on proteostasis. Reversible cysteine-centered modifications, including S-nitrosylation, S-glutathionylation, sulfenylation, and persulfidation, function as physiological redox switches that regulate protein activity, localization, and adaptive stress responses. In contrast, chronic oxidative stress promotes the accumulation of irreversible modifications, including protein carbonylation and advanced oxidation states, which compromise protein stability and increase aggregation propensity. Recent studies further demonstrate that oxidative PTMs can impair multiple components of the proteostasis network, including proteasomal degradation, autophagic flux, and mitochondrial quality-control pathways. These alterations establish a feed-forward cycle in which redox imbalance, defective protein clearance, and protein aggregation progressively reinforce one another (Bibli and Fleming, 2021; Atayik et al., 2021; Jonak et al., 2024; Chahla et al., 2024). Consequently, oxidative PTMs can overwhelm cellular quality-control systems and drive aberrant phase transitions of intrinsically disordered proteins into insoluble, amyloid-like states. Crucially, these PTMs not only accelerate physical misfolding but can also impair the recognition and degradation of pathogenic proteins by both the UPS and autophagic clearance pathways (Davidson and Pickering, 2023).

In addition to oxidative PTMs, growing evidence suggests that metabolic dysfunction can directly influence proteostasis and protein aggregation. Age-associated alterations in amino acid metabolism, lipid homeostasis, cellular bioenergetics, and brain osmolyte composition modify the physicochemical environment in which proteins fold, assemble, and function. Metabolites including myo-inositol, glutamate, choline-containing compounds, N-acetylaspartate (NAA), and creatine have been implicated in regulating protein conformational stability, redox homeostasis, mitochondrial function, and neuronal resilience. Dysregulation of these metabolites has been associated with altered protein folding landscapes, increased aggregation propensity, impaired proteostasis mechanisms, and progression of neurodegenerative pathology. Emerging evidence further indicates that metabolic perturbations can influence liquid–liquid phase separation, mitochondrial quality control, and oxidative stress pathways, thereby acting synergistically with oxidative PTMs to accelerate proteostasis decline and promote the transition from physiological aging to neurodegenerative disease (Kantarci et al., 2000; Ahanger et al., 2025).

This progressive collapse gives rise to a compelling biological paradox: during normal brain aging, there is broad yet functionally silent aggregation across the proteome, whereas neurodegenerative disease states feature a shift toward a more restricted, hyper-toxic set of aggregates that breach pathological thresholds (David et al., 2010; David, 2012; Harel et al., 2024; Chen et al., 2024). The precise biophysical boundaries between the amorphous aggregates seen in normal aging and the highly ordered, propagation-prone species characteristic of disease remain an active, vital area of investigation (Tittelmeier et al., 2020). Compounding this complexity, accumulating evidence indicates that proteostasis extends beyond intracellular quality-control mechanisms. Extracellular chaperones and clearance pathways are critical for maintaining protein homeostasis within the interstitial fluid and synaptic environment, helping to limit the accumulation and spread of toxic oligomeric species (Wilson et al., 2023). Genetic mutations and environmental stressors can further accelerate proteostasis decline by impairing components of these clearance systems or increasing the burden of aggregation-prone proteoforms, thereby reducing network resilience and increasing susceptibility to proteotoxic stress (Hipp et al., 2019; Labbadia and Morimoto, 2015; Yerbury et al., 2016).

In this review, we discuss how age-associated oxidative stress and specific PTMs drive aggregate diversity, shifting the cellular landscape from widespread, benign protein accumulation to targeted neurotoxic assemblies. We compare the broad, heterogeneous protein repertoire of the normally aging central nervous system with the highly ordered, disease-defining Aβ and tau aggregates characteristic of Alzheimer’s disease, highlighting both shared biophysical features and distinct pathological impacts. Furthermore, we examine the structural and functional failure of specific quality control nodes within both the intracellular and extracellular proteostasis networks. Deciphering these underlying mechanisms will provide essential insights into emerging therapeutic approaches—evaluating strategies that either precisely neutralize specific toxic oligomers or globally rejuvenate cellular clearance networks to restore systemic proteome balance (Hipp et al., 2019; Wilson et al., 2023). The conceptual novelty of this framework lies not in any individual component of proteostasis decline, oxidative stress, or protein aggregation, all of which have been extensively studied, but in their integration into a common threshold model. Specifically, we propose that the cumulative burden of oxidative PTMs may represent an important mechanistic driver of threshold crossing, progressively reducing proteostasis capacity while increasing aggregation burden. We further distinguish the broad aggregate diversity characteristic of physiological aging from the aggregate specialization observed in neurodegenerative disease, where a limited number of highly toxic and self-propagating species come to dominate the aggregation landscape. Finally, we incorporate both intracellular and extracellular clearance systems into a unified view of proteostasis collapse (Hassanzadeh et al., 2024).

## 2 Proteostasis network decline in aging

Proteostasis depends on the coordinated balance of protein synthesis, folding, and degradation. With advancing age, this network progressively deteriorates, increasing the accumulation of misfolded and aggregated proteins and enhancing vulnerability to neurodegeneration (Li and Casanueva, 2016; Hipp et al., 2019). Molecular chaperones, including Hsp70 and Hsp90, are central regulators of proteostasis, preventing protein aggregation, promoting proper folding, and directing damaged proteins toward degradation pathways (Hipp et al., 2019; Lackie et al., 2017). Aging is associated with reduced chaperone abundance and activity, accompanied by impaired cellular stress responses, in part due to diminished activation of heat shock factor 1 (HSF1), thereby contributing to proteostasis decline and increased neuronal vulnerability (Li and Casanueva, 2016; Hipp et al., 2019).

In parallel, the ubiquitin–proteasome system (UPS) undergoes both structural and functional deterioration. Aging brains exhibit reduced assembly and activity of the 26S proteasome, together with a relative increase in 20S proteasomes, resulting in less efficient ubiquitin-dependent degradation (Davidson and Pickering, 2023). Oxidative PTMs can further impair ubiquitination pathways and proteasomal substrate recognition, limiting the efficient clearance of damaged proteins (Davidson and Pickering, 2023; Höhn et al., 2020).

Autophagic pathways also decline with age across multiple tissues, and impaired neuronal protein quality control has been linked to increased susceptibility to neurodegeneration (Alfaro et al., 2019; Bourdenx et al., 2021). Chaperone-mediated autophagy (CMA) is particularly affected, owing to age-related reductions in lysosomal LAMP2A levels caused by alterations in lysosomal membrane composition, ultimately resulting in decreased CMA activity (Alfaro et al., 2019).

Together, these age-associated impairments create a state of proteostatic vulnerability in which the balance between protein synthesis, folding, and degradation becomes progressively disrupted. Although this imbalance promotes gradual accumulation of protein aggregates, residual proteostasis capacity often remains sufficient to preserve cellular function and prevent overt neuronal toxicity during normal aging.

## 3 Oxidative post-translational modifications as drivers of protein misfolding

A key mechanistic link between aging and proteostasis decline is the accumulation of oxidative post-translational modifications (PTMs), which directly alter protein structure, stability, and function. In aging tissues, high–molecular weight protein aggregates are enriched in carbonylated proteins, and experimentally induced carbonyl stress in young animals can recapitulate aggregation phenotypes observed during aging, supporting a causal role for oxidative PTMs in protein misfolding and proteostasis failure (Tanase et al., 2016; Dalle-Donne et al., 2003).

Consistent with this concept, chronological aging in yeast is associated with progressive thiol oxidation of proteins involved in translation, folding, and degradation pathways, with highly oxidized species becoming increasingly susceptible to degradation or aggregation (Jonak et al., 2024). Reactive oxygen and nitrogen species (ROS/RNS), generated through mitochondrial dysfunction and metabolic imbalance, modify amino acid residues non-enzymatically, thereby destabilizing the proteome and altering protein function. The age-associated increase in oxidative stress is a conserved phenomenon observed across multiple species and is increasingly recognized as a major contributor to proteostasis decline (Atayik et al., 2021; Jonak et al., 2024).

### 3.1 Types and structural consequences of oxidative PTMs

Oxidative post-translational modifications (PTMs) of cysteine, methionine, and other amino acid residues function as redox “switches” that alter protein conformation, activity, stability, and aggregation behavior. Reversible modifications participate in physiological signaling, whereas irreversible modifications contribute to protein damage and proteotoxic stress. Cysteine residues can undergo a spectrum of oxidative PTMs, including reversible sulfenylation, disulfide bond formation, S-glutathionylation, S-nitrosylation, and persulfidation, as well as more stable sulfinylation and sulfonylation (Shi and Carroll, 2020; Bibli and Fleming, 2021). S-nitrosylation, which involves the covalent attachment of nitric oxide to cysteine residues, plays important regulatory roles under physiological conditions but can contribute to neurodegeneration when dysregulated. For example, S-nitrosylation of proteins such as parkin and protein disulfide isomerase impairs their function and promotes proteotoxic stress (Nakamura and Lipton, 2011). Protein carbonylation, in contrast, represents an irreversible oxidative modification affecting lysine, arginine, proline, and threonine residues that increases hydrophobicity and aggregation propensity (Dalle-Donne et al., 2003). Computational and biophysical studies indicate that even isolated carbonylation events can substantially increase aggregation propensity, whereas extensive carbonyl modification destabilizes protein structure, promotes unfolding, and facilitates self-assembly into higher-order aggregates (Petrov and Zagrovic, 2011).

Methionine residues are likewise highly susceptible to oxidation. Conversion to methionine sulfoxide alters side-chain chemistry and can modify protein structure and function (Chahla et al., 2024). Importantly, methionine sulfoxide reductases can reverse this modification, repairing oxidized proteins and helping to maintain protein function under conditions of oxidative stress and aging (Chahla et al., 2024; Lourenço Dos Santos, et al., 2018).

Importantly, not all oxidative PTMs are inherently pathogenic. Reversible cysteine-centered modifications, including S-nitrosylation, S-glutathionylation, sulfenylation, and persulfidation, serve as dynamic regulators of protein activity, localization, and signaling under physiological conditions and contribute to adaptive stress responses. In contrast, persistent oxidative stress promotes accumulation of irreversible modifications, including protein carbonylation and advanced cysteine oxidation states, which destabilize proteins, disrupt protein–protein interactions, and increase susceptibility to proteotoxic stress. Thus, the biological consequences of oxidative PTMs are highly context dependent, reflecting the balance between adaptive redox signaling and oxidative damage (Bibli and Fleming, 2021; Chahla et al., 2024; Jonak et al., 2024).

### 3.2 PTM-induced phase transitions and aggregation

These PTMs remodel the energy landscape of aggregation-prone proteins by altering charge, hydrophobicity and conformational preferences, thereby lowering effective folding stability and stabilizing misfolded or oligomeric intermediates. Intrinsically disordered proteins such as tau and α-synuclein, which rely on delicate electrostatic and multivalent interactions, are especially sensitive to these PTM-induced shifts (Alquezar et al., 2021; Chiti and Dobson, 2017; Chahla et al., 2024). Oxidative and other PTMs can drive aberrant liquid–liquid phase separation, generating condensates whose material properties gradually shift from liquid-like to gel- or solid-like states, diminishing dynamic exchange and promoting nucleation of fibrillar aggregates. Such liquid-to-solid transitions have been observed for stress granule proteins, FUS, α-synuclein, and oxidized SOD1 and are increasingly viewed as critical intermediates linking soluble intrinsically disordered proteins (IDPs) to irreversible amyloid assemblies (Alquezar et al., 2021; Chahla et al., 2024; Knowles et al., 2014). This process represents a critical intermediate step between soluble proteins and insoluble amyloid aggregates.

### 3.3 Feedback between aggregation and oxidative stress

**FIGURE 1:**
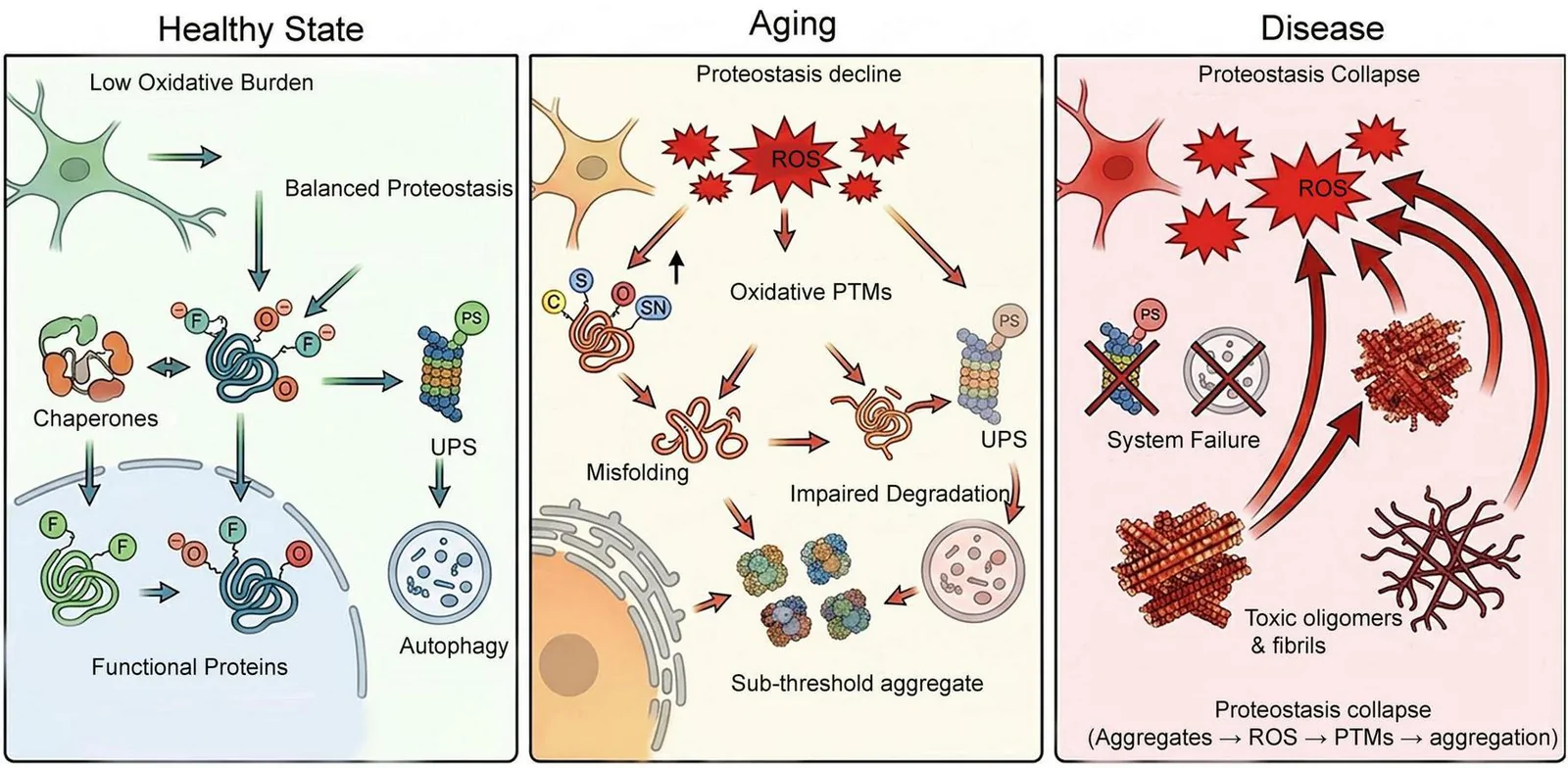
Oxidative post-translational modifications (PTMs) drive proteostasis decline and protein aggregation from aging to neurodegeneration. Schematic representation of the progressive impact of oxidative stress and PTM accumulation on neuronal proteostasis. In the healthy state (left), low levels of reactive oxygen species (ROS) maintain minimal PTM burden and balanced proteostasis, supported by molecular chaperones, the ubiquitin–proteasome system (UPS), and autophagy, thereby preserving functional protein homeostasis. During aging (middle), increased ROS promotes the accumulation of oxidative PTMs, including cysteine oxidation, carbonylation, and S-nitrosylation, leading to protein destabilization, misfolding, and impaired degradation. This results in the formation of heterogeneous, largely sub-threshold aggregates alongside a progressive decline in proteostasis capacity. In neurodegenerative disease (right), excessive PTM burden and sustained oxidative stress drive proteostasis collapse, characterized by failure of clearance systems and accumulation of toxic, structured aggregates, including amyloid-β (Aβ) and tau assemblies. These aggregates further exacerbate oxidative stress, establishing a feed-forward loop that amplifies protein misfolding and aggregation. Schematic showing progression from healthy neurons to aging and neurodegeneration, highlighting increased oxidative stress, accumulation of post-translational modifications, decline in proteostasis systems, and formation of heterogeneous versus toxic amyloid-β and tau aggregates with a feed-forward aggregation loop.

Protein aggregation and oxidative stress are increasingly recognized as mutually reinforcing processes that contribute to neurodegeneration. Rather than acting independently, misfolded proteins and reactive oxygen species (ROS) engage in a self-amplifying cycle that progressively impairs mitochondrial function, weakens antioxidant defenses, and destabilizes proteostasis networks. Aggregation-prone protein species can enhance ROS production through multiple mechanisms, including mitochondrial dysfunction and disrupted cellular homeostasis, whereas oxidative stress promotes additional protein misfolding, aggregation, and sequestration of protective quality-control factors. This reciprocal relationship accelerates proteome instability, proteostasis failure, and cellular dysfunction, thereby linking oxidative PTMs to progressive aggregate accumulation and neurodegenerative pathology (Korovesis et al., 2023; Chico et al., 2026; Figure 1).

## 4 Aggregate diversity in the aging brain reflects broad proteostasis strain

Normal brain aging is increasingly recognized as a state with widespread but heterogeneous protein aggregation, distinct from the focused, hallmark deposits of classic neurodegenerative diseases. Aggregates in aging span many protein classes and often emerge before clear clinical decline. These assemblies are often amorphous and lack the highly ordered β-sheet structures characteristic of amyloids (David et al., 2010; Walther et al., 2015). Proteomic studies in mouse and killifish brains show that aging leads to a broad “age-ggregate” proteome, with 1.3–2.5-fold higher insoluble protein content in old versus young samples. These insoluble species include proteins involved in proteostasis itself (chaperones, proteasome subunits), synaptic function, and metabolism (Cuanalo-Contreras et al., 2023). This suggests a systemic decline in protein quality control rather than selective vulnerability. Importantly, these aggregates are often functionally buffered. Cells may compartmentalize damaged proteins into less toxic deposits or maintain them in partially reversible states through residual chaperone activity (Tyedmers et al., 2010; Saarikangas and Barral, 2015). Nevertheless, this buffering capacity is finite. As aggregate burden accumulates, increasing demands on the proteostasis network gradually exhaust available quality-control resources, reducing system resilience and driving the cell closer to a threshold of proteostatic collapse. Importantly, the distinction between physiological aging and neurodegenerative disease should not be viewed as absolute. Numerous studies have demonstrated that cognitively normal older individuals may accumulate amyloid-β, tau, TDP-43, or Lewy body pathology without overt clinical impairment, suggesting that disease-associated aggregates can emerge along a continuum of age-related proteostasis decline. Conversely, neurodegenerative disorders are not solely characterized by the accumulation of canonical aggregate species but also exhibit widespread alterations in protein solubility, proteostasis network function, and global proteome stability. These observations support a model in which aging and neurodegeneration represent overlapping biological states differing primarily in the degree, composition, and functional consequences of aggregation rather than the complete presence or absence of disease-associated protein assemblies.

In vertebrates, age-dependent aggregation is highly tissue-specific and not explained by expression alone; many aggregates arise from wild-type proteins whose mutations cause human disease, and proteostasis factors (ribosome, chaperones, proteasome) themselves appear in the insoluble fraction (Chen et al., 2024). Ribosome pausing and overload of ribosome-associated quality-control (RQC) pathways can promote aggregation of nascent polypeptides, including proteostasis factors themselves, providing a mechanistic route through which age-related translation stress contributes to proteome instability and proteostasis collapse (Joazeiro, 2019; Stein et al., 2022). These age-related aggregates share detergent insolubility, protease resistance, and amyloid dye binding, and are sequestered into aggresome-like structures, but they span many functional classes rather than a single hallmark protein (Cuanalo-Contreras et al., 2023). In aging killifish and mice, prion-like RNA-binding proteins (e.g., DDX5) form phase-separating condensates that can mature into inactive solid aggregates, suggesting that liquid–solid transitions of disordered proteins are a normal feature of aging brains that can be initially tolerated or even functional before turning detrimental (Harel et al., 2024).

## 5 Aggregate specialization in neurodegenerative disease

Neurodegenerative diseases are distinguished from normal aging by the accumulation of specific misfolded proteins that form ordered amyloid assemblies with prion-like properties. In contrast to the heterogeneous and largely subclinical aggregation observed during normal aging, neurodegenerative diseases are defined by the emergence of highly specific, structurally ordered, and toxic protein aggregates. These assemblies, including amyloid-β (Aβ) and tau in Alzheimer’s disease, exhibit β-sheet-rich amyloid structures and the capacity for seeded propagation (Chiti and Dobson, 2017; Knowles et al., 2014).

Amyloid-β aggregation proceeds through a spectrum of intermediates, where soluble oligomers are now recognized as the principal neurotoxic species, disrupting synaptic signaling and plasticity (Selkoe and Hardy, 2016). Oxidative PTMs further enhance Aβ aggregation and stability, promoting crosslinking and increasing toxicity (Butterfield and Boyd-Kimball, 2019). Tau pathology is driven by a combination of hyperphosphorylation, along with diverse PTMs (glycosylation, ubiquitination, glycation, nitration, oxidation, acetylation), which destabilize microtubule binding and promote aggregation into paired helical filaments and neurofibrillary tangles (Wang and Mandelkow, 2016). These aggregates exhibit prion-like propagation across neural circuits (Goedert, 2015).

Collectively, disease-associated aggregates differ from aging-related assemblies by their structural order, proteome specificity, toxicity, and capacity for spread, marking a transition from global proteostasis stress to targeted pathological processes.

## 6 A threshold model of proteostasis collapse in aging and disease

The progression from physiological aging to neurodegenerative disease can be conceptualized as a threshold-dependent decline in proteostasis network function. During early and midlife, the proteostasis network (PN) buffers protein misfolding through coordinated chaperone, ubiquitin–proteasome system (UPS), and autophagic activities, allowing even inherently aggregation-prone proteins to remain soluble or be efficiently cleared (Hipp et al., 2019; Klaips et al., 2018; Hipp and Hartl, 2024; Labbadia and Morimoto, 2015; Anderton et al., 2026; Chen et al., 2024).

With advancing age, PN capacity progressively declines and insoluble aggregates accumulate across tissues. However, these aggregates often reflect a broadly distributed “metastable subproteome” and may remain compatible with cellular function, particularly when chaperone and proteasome activity are preserved (Hipp et al., 2019; Klaips et al., 2018; Yerbury et al., 2016; Labbadia and Morimoto, 2015). Sustained stress, especially oxidative stress and associated PTMs, further impairs protein folding and degradation, increases the burden of misfolded proteins, and promotes co-aggregation of proteostasis components, thereby reducing PN capacity through a self-reinforcing feed-forward cycle (Hipp and Hartl, 2024; Labbadia and Morimoto, 2015; Kurtishi et al., 2019; Yerbury et al., 2016). As the balance between proteostasis capacity and aggregation load progressively deteriorates, supersaturated proteins such as Aβ, tau, and α-synuclein increasingly evade quality-control mechanisms, nucleate into oligomeric and fibrillar assemblies, and exert a growing influence on the aggregation landscape (Klaips et al., 2018; Hipp and Hartl, 2024; Labbadia and Morimoto, 2015; Yerbury et al., 2016; Chen et al., 2024).

Importantly, the transition from physiological aging to neurodegeneration should not be viewed as a strict binary event. Aggregate accumulation and proteostasis dysfunction likely exist along a continuum, with substantial overlap between aging and disease states. Indeed, many cognitively normal older individuals exhibit varying degrees of amyloid-β, tau, TDP-43, or Lewy body pathology without overt clinical impairment, indicating that disease-associated aggregates can emerge during aging without necessarily crossing pathological thresholds. Conversely, neurodegenerative diseases are increasingly recognized as disorders of widespread proteome instability that extend beyond canonical aggregate species and involve broad alterations in protein solubility, quality-control networks, and cellular proteostasis. These observations suggest that aging and neurodegeneration represent overlapping biological states that differ primarily in the degree, composition, and functional consequences of aggregate accumulation rather than the simple presence or absence of disease-associated proteins.

**FIGURE 2:**
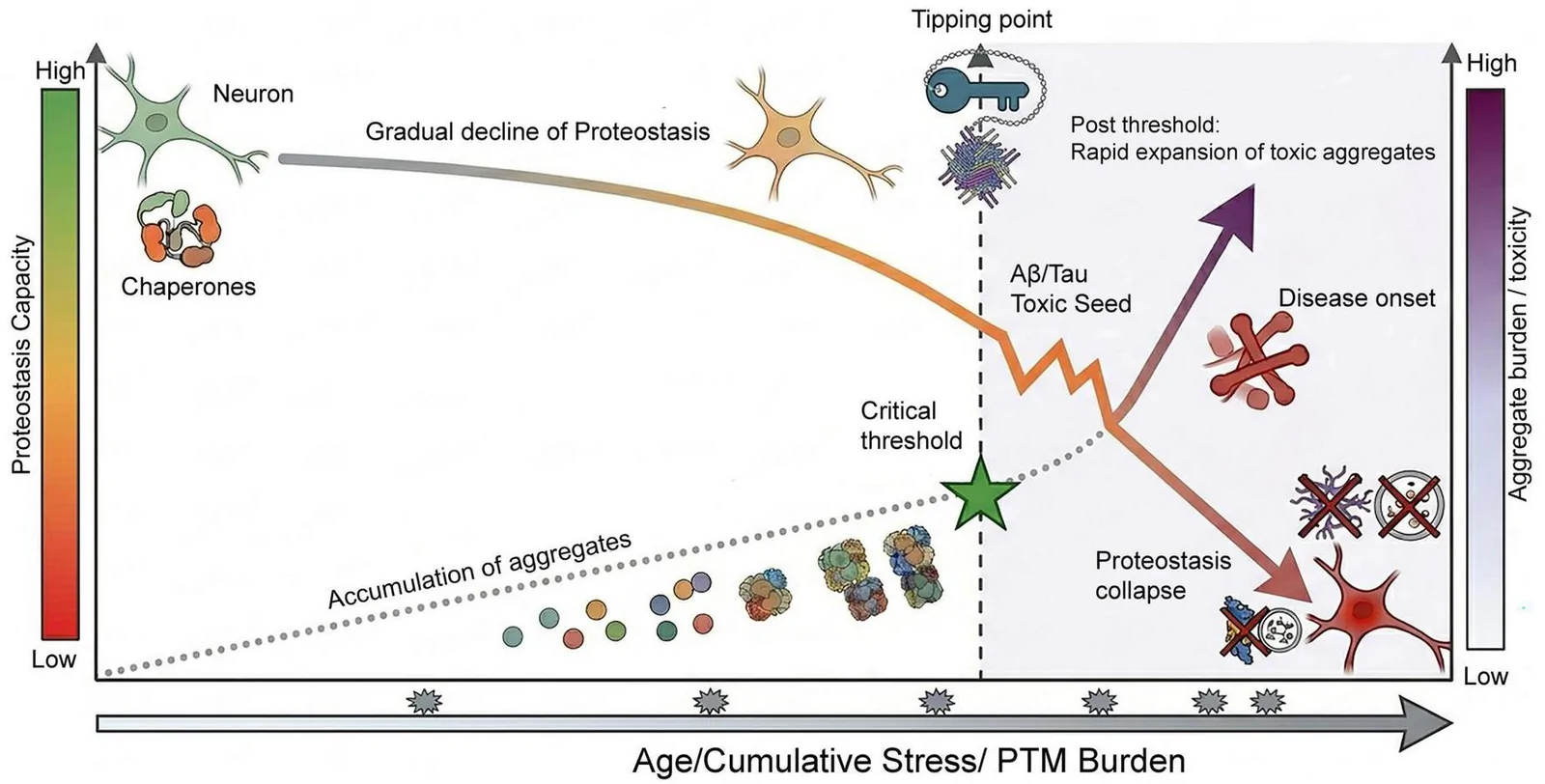
Conceptual threshold model distinguishing aging from neurodegenerative disease. Conceptual model illustrating the transition from physiological aging to neurodegeneration as a progressive, threshold-dependent decline in proteostasis. Proteostasis capacity (left y-axis) gradually decreases with increasing age, cumulative stress, and post-translational modification (PTM) burden (x-axis), while aggregate burden and toxicity (right y-axis) progressively increase. During aging, protein aggregation remains diverse and largely buffered by partially functional proteostasis systems, resulting in the accumulation of heterogeneous, relatively low-toxicity aggregates. Importantly, the boundary between physiological aging and neurodegenerative disease is not absolute, and substantial overlap exists between these states. Many older individuals accumulate disease-associated protein aggregates without overt clinical impairment, whereas neurodegenerative diseases also exhibit widespread proteome instability beyond canonical aggregate species. As proteostasis capacity progressively declines and aggregate burden increases, the system becomes increasingly vulnerable to the emergence and expansion of toxic, self-propagating assemblies, including amyloid-β (Aβ) and tau. The threshold depicted here therefore represents a conceptual transition zone rather than a discrete biological boundary, reflecting both the substantial overlap between physiological aging and neurodegenerative disease and gradual shifts in aggregate burden, cellular resilience, and pathological vulnerability. Beyond this transition zone, aggregate toxicity, impaired clearance, and neuronal dysfunction become increasingly pronounced. Conceptual graph illustrating a threshold-dependent model in which proteostasis capacity gradually declines with age, stress, and post-translational modification burden, while aggregate burden increases. The figure depicts a transition zone rather than a strict boundary between physiological aging and neurodegenerative disease, reflecting overlap between these states. As proteostasis capacity deteriorates, disease-associated aggregates such as amyloid-β and tau become increasingly prominent, accompanied by impaired clearance, increased aggregate toxicity, and heightened neuronal vulnerability.

Accordingly, the threshold model should be viewed as a framework describing gradual shifts in aggregate burden, proteostasis capacity, and cellular vulnerability rather than an abrupt separation between normal aging and neurodegenerative pathology (Figure 2). This transition can be framed as a progressive shift from predominantly diverse and buffered aggregation during physiological aging toward increasingly selective and self-amplifying aggregation in disease, where chronic production of misfolding-prone species and PN insufficiency mutually reinforce one another, ultimately promoting proteostasis collapse and neuronal dysfunction (Klaips et al., 2018; Hipp and Hartl, 2024; Yerbury et al., 2016; Kurtishi et al., 2019; Bourdenx et al., 2021). Importantly, the novelty of this model does not reside in proteostasis decline, oxidative stress, or protein aggregation individually. Rather, it derives from the integration of these processes into a unified framework that identifies cumulative oxidative PTM burden as a potential mechanistic determinant of threshold crossing and emphasizes the transition from broad aggregate diversity in aging to selective aggregate specialization in neurodegenerative disease. This perspective helps explain why widespread age-associated aggregation can remain relatively well-tolerated for prolonged periods, whereas accumulation of specific self-propagating aggregate species is associated with progressive neurodegeneration. Key distinctions between physiological aging and neurodegenerative disease, including proteostasis capacity, aggregate properties, and pathological outcomes, are summarized in Supplementary Table 1.

## 7 Therapeutic implications and future directions

Understanding proteostasis failure as a threshold-dependent process has important therapeutic implications. Once the combined burden of protein misfolding, oxidative stress, and impaired clearance exceeds a critical level, core proteostasis systems—including molecular chaperones, the ubiquitin–proteasome system (UPS), and autophagic pathways—become progressively overwhelmed. This loss of proteostatic capacity promotes secondary aggregation, amplifies proteotoxic stress, and accelerates neuronal dysfunction. Therapeutic strategies targeting individual aggregation-prone proteins, particularly amyloid-β (Aβ) and tau, have demonstrated varying degrees of success in reducing pathological burden and, in some cases, producing measurable clinical benefits. Recent anti-Aβ therapies, including lecanemab and donanemab, have substantially reduced amyloid burden and modestly slowed cognitive decline in individuals with early Alzheimer’s disease (van Dyck et al., 2023; Sims et al., 2023). These findings support the therapeutic value of aggregate-directed approaches. However, such interventions do not fully restore proteostasis and may not address broader network dysfunction, including impaired protein quality control, oxidative stress, mitochondrial dysfunction, and neuroinflammation. Consequently, removal of a single aggregate species may be insufficient to reverse system-wide proteostatic failure.

An emerging therapeutic paradigm therefore combines aggregate-specific interventions with strategies designed to restore proteostasis capacity. Potential approaches include induction of molecular chaperones to improve protein folding and suppress aggregation, enhancement of proteasomal activity to promote clearance of damaged proteins, and modulation of autophagic pathways to facilitate removal of toxic assemblies (Hipp et al., 2019; Bourdenx et al., 2021). In parallel, redox-targeted interventions aimed at limiting oxidative damage and modulating pathological PTMs may reduce formation of aggregation-prone proteoforms. Beyond intracellular mechanisms, enhancement of extracellular clearance pathways—including glymphatic and meningeal lymphatic function—represents a promising strategy for promoting removal of toxic proteins from the aging brain and preserving proteome homeostasis.

Within this framework, oxidative PTMs, particularly cysteine oxidation and related thiol-based modifications, represent important and relatively underexplored nodes linking redox imbalance to protein misfolding, aggregation, and impaired degradation across neurodegenerative diseases, including Alzheimer’s disease, Parkinson’s disease, and amyotrophic lateral sclerosis (ALS). Targeting redox homeostasis and PTM dynamics therefore represents a promising, although still emerging, therapeutic avenue. Rather than broadly suppressing oxidative stress, future strategies may require selective modulation of specific PTMs and redox-sensitive signaling pathways to preserve physiological function while minimizing proteotoxic damage.

Looking forward, integration of proteome-wide aggregation profiling with ultrasensitive PTM-resolved proteomics will be essential for identifying modified protein species that drive toxicity and for defining optimal therapeutic intervention windows. Such approaches may provide a more mechanistic understanding of proteostasis failure and support development of next-generation therapies that combine aggregate-specific targeting with global restoration of proteome stability. Beyond intracellular mechanisms, proteostasis regulation in the central nervous system also depends critically on extracellular clearance pathways, particularly the glymphatic and meningeal lymphatic systems. The glymphatic pathway promotes convective exchange between cerebrospinal fluid (CSF) and interstitial fluid, facilitating clearance of soluble proteins and metabolic waste, including Aβ and tau. Glymphatic function declines with age because of alterations in aquaporin-4 polarization, vascular function, and sleep-dependent clearance dynamics. In parallel, meningeal lymphatic vessels provide an additional route for macromolecule drainage from the brain to peripheral circulation, and dysfunction of these pathways has been associated with pathogenic protein accumulation.

Within the context of proteostasis collapse, impairment of extracellular clearance mechanisms may further lower the threshold for aggregation by increasing residence time of misfolded and aggregation-prone proteins within the interstitial compartment, thereby facilitating oligomer formation, seeding, and intercellular spread. Oxidative stress and PTMs may additionally alter protein solubility and transport properties, further compromising clearance efficiency. Integrating intracellular proteostasis mechanisms with glymphatic and lymphatic function therefore represents an important frontier in understanding aggregate propagation and neurodegenerative disease progression. Therapeutic strategies targeting these coupled clearance systems may provide new opportunities to restore both intracellular protein quality control and extracellular proteome homeostasis.
