# Supplementary material for: Proteostasis breakdown and aggregate diversity in aging and neurodegenerative disease
Source: Front Aging Neurosci. 2026 Aug 11;18:1910154. doi: 10.3389/fnagi.2026.1910154 (PMC13504540; doi:10.3389/fnagi.2026.1910154)
Supplement: Supplementary file 1 [file Table_1.docx]

**Supplementary Table 1. Proteostasis, aggregation properties, and pathological outcomes in aging versus neurodegenerative disease.**

| **Feature** | **Physiological Aging** | **Neurodegenerative Disease** |
| --- | --- | --- |
| Proteostasis capacity | Gradual decline; partially functional | Critically impaired or collapsed |
| Chaperone activity | Reduced expression and stress response | Insufficient; sequestration into aggregates |
| UPS function | Decreased efficiency, reduced 26S activity | Dysfunctional; impaired substrate degradation |
| Autophagy/lysosome | Declining flux; reduced CMA activity | Impaired clearance; lysosomal dysfunction |
| Oxidative stress | Moderately elevated | High and sustained |
| PTM burden | Gradual accumulation (oxidation, carbonylation, S-nitrosylation) | High, dysregulated, drives pathology |
| Aggregate composition | Broad, heterogeneous (metabolic, ribosomal, proteostasis proteins) | Selective (Aβ, tau, α-synuclein, TDP-43) |
| Aggregate structure | Mostly amorphous or low-order assemblies | Highly ordered β-sheet amyloid fibrils |
| Aggregate toxicity | Low to moderate; often buffered | High; toxic oligomers and fibrils |
| Aggregate propagation | Limited; largely intracellular | Prion-like seeding and intercellular spread |
| Phase transition behavior | Reversible or semi-dynamic assemblies | Irreversible liquid-to-solid transitions |
| Cellular impact | Subclinical dysfunction; maintained viability | Synaptic dysfunction and neuronal loss |
| Extracellular clearance (glymphatic/lymphatic) | Mild decline in efficiency | Impaired clearance of toxic species |
| System state | Buffered proteostasis | Proteostasis collapse beyond threshold |
